# NEIGHBORHOOD CHARACTERISTICS AND INEQUALITIES IN SENSORY HEALTH AMONG OLDER ADULTS IN THE UNITED STATES

**DOI:** 10.1093/geroni/igad104.0355

**Published:** 2023-12-21

**Authors:** Alyssa Goldman, Jayant Pinto

**Affiliations:** Boston College, Boston, Massachusetts, United States; University of Chicago, Chicago, Illinois, United States

## Abstract

Sensory function plays a crucial role in older adults’ navigation of the environment, maintenance of personal safety, and in quality of everyday life. The residential neighborhood context is a significant social determinant of later life health and health disparities, but it has been understudied with respect to sensory function. We consider that residential neighborhoods are key sites of social and economic resources, social engagement, and environmental stimulation that could influence sensory health and inequalities in sensory impairment. We use data from 2,251 older adults who were interviewed at Rounds 1 and 2 of the National Social Life, Health and Aging Project in a series of longitudinal random effects models that examine how self-reported and objectively measured neighborhood characteristics are associated with self-rated vision and hearing. We find that older adults who reside in census tracts with higher levels of concentrated disadvantage report significantly worse self-rated vision and hearing compared with older adults who live in areas with less concentrated disadvantage. Residing in a more densely populated tract, however, is associated with better self-rated hearing. Black and Hispanic older adults’ self-rated hearing benefits significantly more from living in a densely populated tract than does White older adults’ self-rated hearing. In cross-sectional models, higher perceptions of neighborhood safety and social ties are associated with better self-rated vision. Our findings prompt a more nuanced understanding of how the social environment affects sensory health and highlight sensory function as a potential underexplored pathway through which neighborhood characteristics shape health disparities in later life.

